# Supplementary material for: Pyruvate Dehydrogenase Kinase 1 inhibition mediated oxidative phosphorylation enhancement in cartilage promotes osteoarthritis progression
Source: BMC Musculoskelet Disord. 2023 Jul 20;24:597. doi: 10.1186/s12891-023-06585-6 (PMC10357736; doi:10.1186/s12891-023-06585-6)
Supplement: Supplementary file 1 — Additional file 1. [file 12891_2023_6585_MOESM1_ESM.zip › BP.Gsea.1653623667859/GOBP_N_GLYCAN_PROCESSING.html]

Details for gene set GOBP\_N\_GLYCAN\_PROCESSING[GSEA]

|  || Dataset | 55457\_collapsed\_to\_symbols.cls.cls #OA\_versus\_normal.cls.cls #OA\_versus\_normal\_repos |
| Phenotype | cls.cls#OA\_versus\_normal\_repos |
| Upregulated in class | OA |
| GeneSet | GOBP\_N\_GLYCAN\_PROCESSING |
| Enrichment Score (ES) | 0.5265212 |
| Normalized Enrichment Score (NES) | 1.6335057 |
| Nominal p-value | 0.02112676 |
| FDR q-value | 0.22234897 |
| FWER p-Value | 1.0 |
Table: GSEA Results Summary

  

Fig 1: Enrichment plot: GOBP\_N\_GLYCAN\_PROCESSING      
 Profile of the Running ES Score & Positions of GeneSet Members on the Rank Ordered List

  

| SYMBOL | TITLE | RANK IN GENE LIST | RANK METRIC SCORE | RUNNING ES | CORE ENRICHMENT || 1 | ST8SIA1 | "ST8 alpha-N-acetyl-neuraminide alpha-2,8-sialyltransferase 1 [Source:HGNC Symbol;Acc:HGNC:10869]" | 133 | 0.873 | 0.1321 | Yes |
| 2 | MGAT4A | "alpha-1,3-mannosyl-glycoprotein 4-beta-N-acetylglucosaminyltransferase A [Source:HGNC Symbol;Acc:HGNC:7047]" | 657 | 0.604 | 0.1900 | Yes |
| 3 | MAN2A1 | mannosidase alpha class 2A member 1 [Source:HGNC Symbol;Acc:HGNC:6824] | 1069 | 0.501 | 0.2399 | Yes |
| 4 | GANAB | glucosidase II alpha subunit [Source:HGNC Symbol;Acc:HGNC:4138] | 1267 | 0.464 | 0.3004 | Yes |
| 5 | MAN1C1 | mannosidase alpha class 1C member 1 [Source:HGNC Symbol;Acc:HGNC:19080] | 1275 | 0.463 | 0.3753 | Yes |
| 6 | FUT8 | fucosyltransferase 8 [Source:HGNC Symbol;Acc:HGNC:4019] | 1565 | 0.418 | 0.4211 | Yes |
| 7 | PRKCSH | protein kinase C substrate 80K-H [Source:HGNC Symbol;Acc:HGNC:9411] | 1679 | 0.402 | 0.4779 | Yes |
| 8 | ST8SIA4 | "ST8 alpha-N-acetyl-neuraminide alpha-2,8-sialyltransferase 4 [Source:HGNC Symbol;Acc:HGNC:10871]" | 1850 | 0.379 | 0.5265 | Yes |
| 9 | GNPTAB | N-acetylglucosamine-1-phosphate transferase subunits alpha and beta [Source:HGNC Symbol;Acc:HGNC:29670] | 2648 | 0.285 | 0.5112 | No |
| 10 | MAN1B1 | mannosidase alpha class 1B member 1 [Source:HGNC Symbol;Acc:HGNC:6823] | 3447 | 0.207 | 0.4831 | No |
| 11 | ST8SIA2 | "ST8 alpha-N-acetyl-neuraminide alpha-2,8-sialyltransferase 2 [Source:HGNC Symbol;Acc:HGNC:10870]" | 4153 | 0.142 | 0.4515 | No |
| 12 | MAN1A2 | mannosidase alpha class 1A member 2 [Source:HGNC Symbol;Acc:HGNC:6822] | 4272 | 0.131 | 0.4637 | No |
| 13 | MGAT4B | "alpha-1,3-mannosyl-glycoprotein 4-beta-N-acetylglucosaminyltransferase B [Source:HGNC Symbol;Acc:HGNC:7048]" | 5956 | -0.002 | 0.3333 | No |
| 14 | ST8SIA5 | "ST8 alpha-N-acetyl-neuraminide alpha-2,8-sialyltransferase 5 [Source:HGNC Symbol;Acc:HGNC:17827]" | 6565 | -0.047 | 0.2938 | No |
| 15 | ST8SIA3 | "ST8 alpha-N-acetyl-neuraminide alpha-2,8-sialyltransferase 3 [Source:HGNC Symbol;Acc:HGNC:14269]" | 10046 | -0.326 | 0.0768 | No |
| 16 | MAN1A1 | mannosidase alpha class 1A member 1 [Source:HGNC Symbol;Acc:HGNC:6821] | 10207 | -0.341 | 0.1201 | No |
| 17 | MAN2A2 | mannosidase alpha class 2A member 2 [Source:HGNC Symbol;Acc:HGNC:6825] | 11772 | -0.545 | 0.0876 | No |
Table: GSEA details [plain text format]

  

Fig 2: GOBP\_N\_GLYCAN\_PROCESSING      
 Blue-Pink O' Gram in the Space of the Analyzed GeneSet

  

Fig 3: GOBP\_N\_GLYCAN\_PROCESSING: Random ES distribution      
 Gene set null distribution of ES for **GOBP\_N\_GLYCAN\_PROCESSING**
